# A New Look at the Coefficients of a Reciprocal Generating Function

**DOI:** 10.1155/2014/613947

**Published:** 2014-08-28

**Authors:** Wiktor Ejsmont

**Affiliations:** Department of Mathematics and Cybernetics, Wroclaw University of Economics, Komandorska 118/120, 53-345 Wroclaw, Poland

## Abstract

We study a special property of free cumulants. We prove that coefficients of a reciprocal generating function correspond to “free cumulants with the first two elements in the same block.”

## 1. Introduction

The original motivation for this paper comes from a desire to understand the results on free probability method in the book of Nica and Speicher [1]. Free probability is a mathematical theory that studies noncommutative random variables. The freeness or free independence property is the analogue of the classical notion of independence, and it is connected with free products. This theory was initiated by Dan Voiculescu around 1986 in order to attack the free group factors isomorphism problem, an important unsolved problem in the theory of operator algebras (see [2]).

It is natural to study relations between classical analysis and free probability. We will present a theorem which gives us a new relation between reciprocal generating function and free cumulants. The free cumulant (introduced by Speicher) [3] plays a major role in the free probability theory. It is related to the lattice of noncrossing partitions of the set {1,…, *n*} in the same way in which the classic cumulant functional is related to the lattice of all partitions of that set.

Since these beginnings of free probability theory have evolved into a theory with a lot of links in quite different fields, in particular, there exists a combinatorial facet: main aspects of free probability theory can be considered the combinatorics of noncrossing partitions. It is worthwhile to mention the work of [4], where authors introduced the so-called Bercovici-Pata bijection. This bijection, which we denote by Λ, is a correspondence between the probability measures on the real line that are infinitely divisible with respect to the classical convolution ∗ and the ones which are infinitely divisible with respect to the free convolution ⊞. This mapping has several useful algebraic and topological properties and preserves the properties of cumulant, that is, determines the uniqueness between classical and free cumulant. Moreover, by suitable definition of the free cumulant transform, the connection between the free and classical Lévy-Khintchine representations of a probability law in *ID*(∗) and its counterpart Λ in *ID*(⊞) is determined simply by *μ* and Λ(*μ*) having the same characteristic triplet (classical and free, resp.). The main aim of the paper is to show new property of free cumulants; that is, we will show that coefficients of a reciprocal generating function correspond to “cumulant with the first two elements in the same block.” This topic has not been extensively studied in the past. The only result of which I am aware is Wright's [5]. Wright gave an asymptotic formula for the coefficient in some special case of power series. Recently, this topic has also been studied in [6], where the concept of composita of reciprocal generating function is used for obtaining a unique triangle, when only the generating function for the central coefficients of that triangle is known. In this paper, we present a new method using free cumulants to compute coefficients of a reciprocal generating function.

## 2. Free Cumulants and Indication

In this section, we provide a short and self-contained summary of the basic definitions and facts needed for our study. In free probability, we assume that our probability space is a von Neumann algebra *A* with a normal faithful tracial state *τ* : *A* → *C*; that is, *τ*(·) is linear and weak*-continuous and *τ*(*XY*) = *τ*(*YX*), *τ*(*I*) = 1, *τ*(XX*) ≥ 0, and *τ*(XX*) = 0 imply *X* = 0 for all *X*, *Y* ∈ *A*. A (noncommutative) random variable *X* is a self-adjoint (*X* = X*) element of *A*. Below, we introduce the concept of noncrossing cumulants without using this abstract object.

**Definition 1 .**

Let *π* = {*V*_1_,…, *V*_*p*_} be a partition of the linear ordered set 1,…, *n*; that is, the *V*_*i*_ ≠ *∅* are ordered and disjoint sets whose union is {1,…, *n*}. Then *π* is called noncrossing if *a*, *c* ∈ *V*_*i*_ and *b*, *d* ∈ *V*_*j*_  with *a* < *b* < *c* < *d* which implies *i* = *j*. The sets *V*_*i*_ ∈ *π* are called blocks. We will denote the set of all noncrossing partitions of the set {1,…, *n*} by NC(*n*).

Now we can define the free cumulants by induction. An important technical tool is a formula, that is, (2), to factorize in a product according to the block structure of noncrossing partition. This formula is actually at the basis of many of our forthcoming results in this paper and allows elegant proofs of many statements.

**Definition 2 .**

Let *a*_1_, *a*_2_,… be a sequence of *C*. The free (noncrossing) cumulants are the sequence *R*_*k*_≔*R*_*k*_(*a*_1_,…, *a*_*k*_) defined by the recursive formula

(1) $$\begin{array}{c} a_{n}=\underset{\nu \in \text{NC}(n)}{\sum }R_{\nu }\left(a_{1},a_{2},\ldots ,a_{n}\right), \end{array}$$

where

(2) $$\begin{array}{c} R_{\nu }\left(a_{1},a_{2},\ldots ,a_{n}\right)≔\Pi_{B\in \nu }R_{\left|B\right|}\left(a_{i}:i\in B\right) \end{array}$$

and NC(*n*) is the set of all noncrossing partitions of {1,2,…, *n*} (see [1, 7]).

**Example 3 .**

For *n* = 3, we have

(3) NC(3)={{(1,2),(3)},{(1,3),(2)},{(2,3),(1)},  {(1,2,3)},{(1),(2),(3)}},a3=∑ν∈NC(3)Rν(a1,a2,a3)=R2(a1,a2)R1(a3)+R2(a1,a3)R1(a2) +R2(a2,a3)R1(a1)+R3(a1,a2,a3) +R1(a1)R1(a2)R1(a3).

**Definition 4 .**

The ordinary generating function of a sequence (*a*_0_, *a*_1_, *a*_2_,…), where *a*_*i*_ ∈ *C*, is

(4) $$\begin{array}{c} G\left(z\right)=\overset{\infty }{\underset{i=0}{\sum }}a_{i}z^{i}. \end{array}$$

In this paper, we assume that *a*_0_ ≠ 0 above series is convergent for sufficiently small *z*.

The following lemma is a sequence version of Lemma  2.3 in [9] with *k* = 1 (the proof is also similar).

**Lemma 5 .**

Let *a*_1_, *a*_2_,… be a sequence of *C*. Then

(5) $$\begin{array}{c} a_{n}=\overset{n-2}{\underset{i=0}{\sum }}a_{i}\underset{\nu \in NC^{\prime \prime }\left(n-i\right)}{\sum }R_{\nu }\left(a_{1},\ldots ,a_{n-i}\right)+a_{1}a_{n-1}, \end{array}$$

where

- *n*  is positive integer greater than two;
- *a*_0_ = 1;
- *NC*′′(*n*) is the set of all noncrossing partitions of {1,2,…, *n*} with the first two elements in the same block.

**Proof**

At first, we will consider partitions with singleton 1; that is, *π* ∈ NC(*n*) and *π* = {*V*_1_,…, *V*_*k*_}, where *V*_1_ = {1}. It is clear that the sum over all noncrossing partitions of this form corresponds to the term *a*_1_ *a*_*n*−1_.

**Figure 1:**
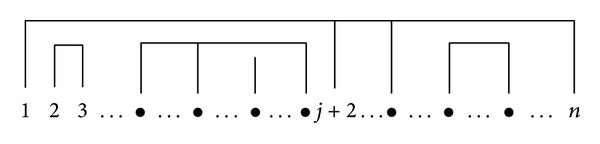
The main structure of noncrossing partitions of {1,2, 3,…, *n*} with the first and *j* + 2 element in the same block.

On the other hand, for such partitions *ν* ∈ NC(*n*), let *k* = *k*(*ν*)∈{3,4,…, *n*} denote the most-left element of the block containing 1. This decomposes NC(*n*) into the *n* − 1 classes NC_*j*_′′(*n*) = {*ν* ∈ NC(*n*) : *k*(*ν*) = *j* + 2}, *j* = 0,1, 2,…, *n* − 2. The set NC_*j*_′′(*n*) can be identified with the product NC(*j*) × NC′′(*n* − *j*) for *j* > 0 and NC_0_′′(*n*) = NC′′(*n*). Indeed, the blocks of *ν* ∈ NC_*j*_′′(*n*), which partition the elements {2,3, 4,…, *j* + 1}, can be identified with an appropriate partition in NC(*j*), and (under the additional constraint that the first two elements 1, *j* + 2 are in the same block) the remaining blocks, which partition the set {1, *j* + 2, *j* + 3,…, *n*}, can be uniquely identified with a partition in NC′′(*n* − *j*). The above situation is illustrated in Figure 1. This gives the formula

(6) an=∑ν∈NC(n)Rν(a1,…,an)=∑i=0n−2 ∑ν∈NC(i)Rν(a1,…,ai) ×∑ν∈NC′′(n−i)Rν(a1,…,an−i)+a1an−1=∑i=0n−2ai∑ν∈NC′′(n−i)Rν(a1,…,an−i)+a1an−1,

which proves the lemma.

**Definition 6 .**

Let *a*_0_, *a*_1_, *a*_2_,…∈*C* and *a*_0_ ≠ 0. We define

(7) cn(2)≔cn(2)(a0,a1,a2,…,an)=∑ν∈NC′′(n)Rν(a1a0,a2a0,…,ana0)

for *n* ≥ 2 and the functions (power series)

(8) $$\begin{array}{c} C^{\left(2\right)}\left(z\right)=\overset{\infty }{\underset{n=2}{\sum }}c_{n}^{\left(2\right)}z^{n} \end{array}$$

for sufficiently small |*z*| < *ϵ* and *z* ∈ *C*. This series is convergent because we consider only (*a*_1_, *a*_2_,…) such that |∑_*n*=1_^*∞*^ *a*_*n*_ *z* ^*n*^| < *∞*. This implies that ∑_*n*=2_^*∞*^ *c*_*n*_^(2)^ *z* ^*n*^ is convergent and the proof of this fact follows from the main result.

## 3. The Main Result

The following is our main results of the paper.

**Theorem 7 .**

Let *a*_0_, *a*_1_, *a*_2_,… be a sequence of *C* (*a*_0_ ≠ 0) with the generating function *G*(*z*) = *a*_0_ + ∑_*i*=1_^*∞*^ *a*_*i*_ *z* ^*i*^, and then

(9) $$\begin{array}{c} \frac{1}{G\left(z\right)}=\frac{1}{a_{0}}\left(1-C^{\left(2\right)}\left(z\right)-\frac{a_{1}}{a_{0}}z\right). \end{array}$$

In other words, sequence (1/*a*_0_, −*a*_1_/*a*_0_^2^, −*c*_2_^(2)^/*a*_0_, −*c*_3_^(2)^/*a*_0_,…) is the generating function of 1/*G*(*z*).

**Proof**

It is clear from Lemma 5 that we have

(10) 1a0G(z)−1=1a0∑n=1∞anzn=(a1a0z+∑n=2∞ana0zn)=a1a0z +∑n=2∞[∑i=0n−2aia0cn−i(2)(a0,a1,a2,…,an−i)+a1a0an−1a0]zn=a1a0z+∑n=2∞ ∑i=0n−2aia0zicn−i(2)(a0,a1,a2,…,an−i)zn−i +a1a0z∑n=2∞an−1a0zn−1=∑n=2∞ ∑i=0n−2aia0zicn−i(2)(a0,a1,a2,…,an−i)zn−i +a1a0z[1+∑n=2∞an−1a0zn−1]=1a0G(z)C(2)(z)+a1a02zG(z),

where *a*_0_ = 1, and, in the last equality, we use the Cauchy product of two series. This proves the Theorem.

**Corollary 8 .**

If *a*_0_ = 1, then one gets

(11) $$\begin{array}{c} \frac{1}{G\left(z\right)}=1-C^{\left(2\right)}\left(z\right)-a_{1}z. \end{array}$$

**Example 9 .**

The *n*th Catalan number is given directly in terms of binomial coefficients by $\left(1/\left(n+1\right)\right)\left(\begin{array}{c} 2n\\ n \end{array}\right)$. Let *b*_0_ = 1 and

(12) $$\begin{array}{c} b_{n}=\{\begin{array}{cc} 0, & \text{for}\;n\;\text{odd},\\ \frac{1}{n/2+1}\left(\begin{array}{c} n\\ \frac{n}{2} \end{array}\right), & \text{for}\;n\;\text{even}. \end{array} \end{array}$$

Then the *b*_*n*_ is the *n*th moment of Wigner semicircle law of mean 0 and variance 1 (see [10]). The free cumulant of *b*_*n*_ is the number of noncrossing partitions of the set {1,…2*n*} in which every block is of size 2 (see, e.g., [3, 8, 11–13]); that is, *b*_*n*_ = ∑_*ν*∈NC(*n*)_ *R*_*ν*_(*b*_1_, *b*_2_,…, *b*_*n*_), where *R*_*ν*_(*b*_1_, *a*_2_,…, *b*_*n*_)≔Π_*B*∈*ν*_ *R*_|*B*|_(*b*_*i*_ : *i* ∈ *B*) and

(13) $$\begin{array}{c} R_{n}\left(b_{1},b_{2},\ldots ,b_{n}\right)=\{\begin{array}{cc} 1, & \text{for}\;n=2,\\ 0, & \text{otherwise}. \end{array} \end{array}$$

Thus, we get

(14) cn(2)=cn(2)(1,b1,b2,…,bn)={0,for n odd,1(n−2)/2+1(n−2n−22),for n even,1G(z)=1−∑i=2∞zncn(2)=1−z2G(z).

**Example 10 .**

In random matrix theory, the Marčenko-Pastur distribution, or Marčenko-Pastur law, describes the asymptotic behavior of singular values of large rectangular random matrices. The theorem is named after Ukrainian mathematicians Marčenkoand Pastur who proved this result in 1967 (see [7]). Moments of the Marčenko-Pastur law (with rate 1 and jump size 1) are

(15) $$\begin{array}{c} d_{n}=\overset{n-1}{\underset{i=0}{\sum }}\frac{1}{i+1}\left(\begin{array}{c} n\\ i \end{array}\right)\left(\begin{array}{c} n-1\\ i \end{array}\right). \end{array}$$

Then, *d*_*n*_ have free cumulants, which are constant; that is, *R*_*n*_(*d*_1_, *d*_2_,…, *d*_*n*_) = 1 (see [8, 10]). Thus, if *d*_0_ = 1, then

(16) $$\begin{array}{c} c_{n}^{\left(2\right)}=c_{n}^{\left(2\right)}\left({1,d}_{1},d_{2},\ldots ,d_{n}\right)=d_{n-1}. \end{array}$$

Indeed, the first block of *ν* ∈ NC′′(*n*) (which contains {1,2}) can be identified with an appropriate block in NC(*n* − 1) (i.e., which contains {1}) because *R*_*n*_ are constant. Thus, we get

(17) 1G(z)=1−z−∑i=2∞zncn(2)=1−z−z(G(z)−1)=1−zG(z).

*Open Problem*. It would be worth to show whether Theorem 7 is true for noncommutative *c*-free cumulants (for more details, see [14]).

## References

[1] Nica A, Speicher R (2006). *Lectures on the Combinatorics of Free Probability*.

[2] Voiculescu DV, Dykema KJ, Nica A (1992). A noncommutative probability approach to free products with applications to random matrices, operator algebras and harmonic analysis on free groups. *Free Random Variables*.

[3] Speicher R (1994). Multiplicative functions on the lattice of noncrossing partitions and free convolution. *Mathematische Annalen*.

[4] Bercovici H, Pata V (1999). Stable laws and domains of attraction in free probability theory. *Annals of Mathematics. Second Series*.

[5] Wright EM (1966). Coefficients of a reciprocal generating function. *The Quarterly Journal of Mathematics*.

[6] Kruchinin D, Kruchinin V (2012). A method for obtaining generating functions for central coefficients of triangles. *Journal of Integer Sequences*.

[7] Marčenko VA, Pastur LA (1967). Distribution of eigenvalues for some sets of random matrices. *Mathematics of the USSR-Sbornik*.

[8] Ejsmont W (2013). Noncommutative characterization of free Meixner processes. *Electronic Communications in Probability*.

[9] Ejsmont W (2014). Characterizations of some free random variables by properties of conditional moments of third degree polynomials. *Journal of Theoretical Probability*.

[10] Bożejko M, Bryc W (2006). On a class of free Lévy laws related to a regression problem. *Journal of Functional Analysis*.

[11] Anshelevich M (2003). Free martingale polynomials. *Journal of Functional Analysis*.

[12] Bożejko M, Leinert M, Speicher R (1996). Convolution and limit theorems for conditionally free random variables. *Pacific Journal of Mathematics*.

[13] Ejsmont W (2012). Laha-Lukacs properties of some free processes. *Electronic Communications in Probability*.

[14] Ejsmont W (2014). New characterization of two-state normal distribution. *Infinite Dimensional Analysis, Quantum Probability and Related Topics*.

